# Interplay of Transforming Growth Factor-Beta 1 and 3 in the Pathogenesis of Oral Submucous Fibrosis and Its Malignant Transformation: An Immunohistochemical Study

**DOI:** 10.7759/cureus.42412

**Published:** 2023-07-24

**Authors:** Shivani Bansal, Treville Pereira, Rajiv S Desai, Abinashi Jena, Poorvashree P Bobade, Madhura Patil

**Affiliations:** 1 Department of Oral Pathology and Microbiology, Nair Hospital Dental College, Mumbai, Mumbai, IND; 2 Department of Oral Pathology and Microbiology, School of Dentistry, D. Y. Patil University, Mumbai, IND

**Keywords:** transforming growth factor-beta, oral squamous cell carcinoma, oscc, tgf-β3, tgf-β1, tgf-β, osf, oral submucous fibrosis

## Abstract

Introduction

Oral submucous fibrosis (OSF) is a chronic and potentially malignant oral condition that poses a significant public health issue due to its insidious nature. Transforming growth factor-beta (TGF-β) is a key player in the pathogenesis of OSF and is responsible for fibrosis. This study aims to investigate the relationship between the expression of TGF-β1 and TGF-β3 in OSF and its malignant transformation by using immunohistochemistry.

Materials and method

The present study comprised of 120 formalin-fixed paraffin-embedded tissue samples, which included 20 normal oral mucosa (NOM), 80 OSF (20 each of stage 1- 4), and 20 oral squamous cell carcinoma (OSCC) (10 each of OSCC with and without OSF), and were stained for TGF-β1 and TGF-β3 by immunohistochemistry. Data were analyzed using R software version 4.1.2, GraphPad Prism 9.3.1 (GraphPad Software, San Diego, CA, USA) and Excel (Microsoft Corp., Redmond, WA).

Results

TGF-β1 immunoexpression was negative in NOM with no significant difference among OSF and OSCC (with or without OSF). TGF-β3 was significantly higher in OSCC (with or without OSF) than in OSF, and no significant difference was noted between OSF and NOM and between OSCC and NOM. A significant increase was seen in TGF-β3 compared to TGF-β1 in NOM, OSF (stage 1- 4), and OSCC with and without OSF.

Conclusion

TGF-β3 has higher immunoexpression levels than TGF-β1 in NOM, OSF, and OSCC. We speculate that quantitative or qualitative TGF- β3 may be inadequate to prevent or attenuate fibrosis in OSF patients. There is also a modicum of probability that TGF-β3 has a preventive rather than causative role in OSF pathogenesis. The role of TGF-β3 in OSF needs further clarification.

## Introduction

Oral submucous fibrosis (OSF) is a chronic progressive, potentially malignant disorder characterized by juxta-epithelial hyalinization followed by generalized submucosal fibrosis and epithelial atrophy, which leads to difficulty in mouth opening and has a malignant transformation rate of 7-13%. OSF is a major concern worldwide, especially in South and Southeast Asia [1,2]. The pathogenesis of OSF is obscure and believed to be multifactorial. It includes areca nut chewing, ingestion of chilies, genetic and immunologic processes, nutritional deficiencies, and other factors [2].

The exact mechanism of submucosal deposition of collagen in OSF is still unknown [1]. However, research suggests that transforming growth factor-beta (TGF-β) is a key factor in the development of fibrosis, as it triggers an increase in collagen production and a decrease in matrix degradation [1,3,4]. TGF-β is a multifunctional cytokine that belongs to the transforming growth factor superfamily and includes three structurally similar mammalian isoforms (TGF-β1, TGF-β2, TGF-β3). They exert nearly identical effects grouped into three broad areas: modulation of inflammatory cell function, growth inhibition and differentiation, and control of extracellular matrix production [3]. TGF-β1 and TGF-β2 display potent fibrotic activity, while TGF-β3 has a more anti-fibrotic effect [3,5]. Additionally, TGF-β plays a critical role in tumor formation and progression [3,6], but the role and contribution of TGF-β1 and TGF-β3 in oral squamous cell carcinoma (OSCC) with OSF remains unclear.

Our study aims to investigate the relationship between the expression of TGF-β1 and TGF-β3 in different stages of OSF and OSCC, with or without OSF, compared to healthy controls by immunohistochemistry (IHC). We evaluated the possible role of TGF-β1 and TGF-β3 in the malignant transformation of OSF. This information could provide valuable insight into the biological behavior of these lesions, thereby aiding in therapeutic intervention.

## Materials and methods

The present study was approved by the Institutional Ethics Committee (EC-PhD-03/PATHO-03/2020). A total of 120 formalin-fixed paraffin-embedded tissue (FFPE) blocks were retrieved from the archives of the Department of Oral Pathology & Microbiology, Nair Hospital Dental College, Mumbai, India. The study included 80 diagnosed cases of OSF divided into four stages (20 of each stage), 10 cases of well-differentiated OSCC without OSF, graded according to the Broders classification [7], 10 cases of OSCC with OSF, and 20 samples of normal oral mucosa (NOM) obtained from the surgical site of third molar impaction of healthy individuals without any deleterious habit. It is well documented in the literature that there is no correlation between clinical and histopathological findings in OSF. Therefore, the staging of OSF cases was performed according to the degree of mouth opening by Lai et al.: stage 1, mouth opening greater than 35 mm; stage 2, mouth opening between 30 and 35 mm; stage 3, mouth opening between 20 and 30 mm; and stage 4, mouth opening less than 20 mm [8].

As a tertiary health care center, OSF patients reporting to our department are from low-income backgrounds and distant locations. Keeping track of these patients can be challenging due to low response rates and difficulty reaching those who have relocated or changed contact information. Additionally, following up on potential malignant cases is daunting due to limited resources and a diverse patient population. To overcome this challenge, we have studied cases where both OSF and OSCC were present synchronously. We did not include transformed OSF cases.

Immunohistochemical staining

Primary anti-TGF-β1 (rabbit monoclonal, ab215715; Abcam, 1:100) vial (100 μl) and anti-TGF-β3 (rabbit polyclonal, ab15537; Abcam, 1:200) antibodies were used to perform immunohistochemical staining on 3-μm-thick sections obtained from FFPE tissue blocks using the BioGenex Super Sensitive Polymer-HRP IHC detection system (BioGenex, Fremont, CA). Horseradish peroxidase was used as an enzyme label, and diaminobenzidine (DAB) was used as a substrate substrate to locate the antibody binding. The sections were counterstained with Mayer’s hematoxylin, mounted with ibutylphthalate polystyrene xylene (DPX) and viewed under research microscope (Optika, Ponteranica, Italy). Positive controls for TGF-β1 and TGF-β3 were human spleen (megakaryocytes) and placenta, respectively. Cytoplasm showing a clear brown color was considered as a positive immunoreaction. The representative fields were randomly selected in each sample for semi-quantitative analysis and evaluated for TGF-β1 and TGF-β3 immunostaining in epithelial and connective tissue. To ensure an unbiased analysis, we conducted a double-blinded study. Two experienced oral pathologists supervised the process and resolved any disagreements through discussion. If a conflict persisted, a third researcher's judgment was deemed final. The scores given by both observers were averaged and recorded for data analysis. The staining intensity and proportion of cell staining positively were scored using criteria given by Reiner et al. [9]. Staining intensity was scored as 0 (no staining), 1 (× 400), 2 (× 100), and 3 (× 40). The total proportion of cell staining was calculated as 0 (no cell staining), 1 (1-5% stained cells), 2 (6-25% stained cells), 3 (26-50% stained cells), and 4 (>50% stained cells) under high-power magnification (×400). The results of intensity and proportion scores were added to obtain a “quick score.” A score of 2 or 3 was considered low, 4 or 5 was intermediate, and 6 or 7 was high.

Statistical analysis

The data were analyzed using R software version 4.1.2, GraphPad Prism 9.3.1 (San Diego, CA, USA), and Excel. Categorical variables were given in the form of a frequency table. Continuous variables were given in mean ± SD or median (min, max) form. The Mann-Whitney U test was used to compare the distributions over growth factors. The Kruskal-Wallis test was used to compare the distribution of variables across groups. Dunn’s test was used as post hoc analysis. Confidence intervals were set at 95%, and a p-value of ≤ .05 was considered statistically significant.

## Results

The present study included 80 OSF subjects, with 63 (78.8%) male and 17 (21.2%) female, with a gender ratio of 3.7:1. Age range was 16-65 years, with an average age of 40.5 years. Histopathological examination of OSF samples showed no signs of dysplasia.

TGF-β1 expression in NOM, OSF (stage 1 to stage 4), and OSCC with and without OSF

In the epithelium, TGF-β1 expression was significantly higher in OSCC (with or without OSF) than in OSF (p < 0.05) and was negative in NOM. In OSF, expression levels increased in stage 3 compared to other stages (p < 0.01), and stage 2 had higher expression levels than stages 1 and 4 (p < 0.05). No significant difference was found between OSF stage 1 and 4 and OSF stage 3 and OSCC with or without OSF (p > 0.05). In connective tissue, TGF-β1 expression was significantly higher in OSF than OSCC (with or without OSF) and OSCC with OSF (p < 0.05). OSF stage 3 showed a significant increase compared to other stages and OSCC with OSF (p < 0.05). There was no significant difference between OSF stage 3 and OSCC without OSF (p > 0.05) (Table 1).

**Table 1:** Comparison of TGF-β1 expression in epithelium and the connective tissue for NOM, OSF stage 1, OSF stage 2, OSF stage 3, OSF stage 4, and OSCC with and without OSF MW, Mann-Whitney U test; NOM, normal oral mucosa; K, Kruskal-Wallis test; OSCC, oral squamous cell carcinoma; OSF, oral submucous fibrosis; TGF-β1, transforming growth factor-beta 1 *Indicates statistical significance

Groups	Epithelium	Connective tissue	p-value
NOM	0 ± 0	0 ± 0	-
	0 (0, 0)	0 (0, 0)	
OSF stage 1	0.35 ± 1.09	2.3 ± 1.98	<0.001^MW^*
	0 (0, 4)	3 (0, 5)	
OSF stage 2	1.8 ± 1.01	1.95 ± 1.54	0.305^MW^
	2 (0, 3)	3 (0, 4)	
OSF stage 3	3.55 ± 1.28	3.65 ± 0.88	0.481^MW^
	3 (2, 6)	4 (2, 5)	
OSF stage 4	0.6 ± 1.23	1.75 ± 1.86	0.0253^MW^*
	0 (0, 3)	1 (0, 4)	
OSCC	2.7 ± 2.26	2.4 ± 2.22	0.756^MW^
	2.5 (0, 6)	3 (0, 6)	
OSCC + OSF	2.4 ± 1.9	1.5 ± 1.72	0.268^MW^
	2.5 (0, 5)	1 (0, 4)	
p-value	<0.001^K^*	<0.001^K^*	-

**Figure 1:**
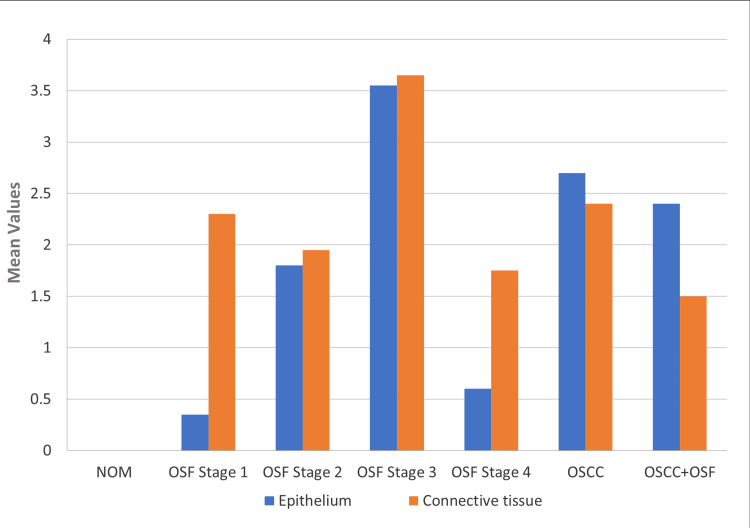
Mean quick score comparison of TGF-β1 expression in epithelium and the connective tissue for NOM, OSF stage 1, OSF stage 2, OSF stage 3, OSF stage 4, OSCC, and OSCC with OSF NOM, normal oral mucosa; OSCC, oral squamous cell carcinoma; OSF, oral submucous fibrosis; TGF-β1, transforming growth factor-beta 1

Additionally, there was a significant increase in connective tissue as compared to epithelium in OSF (p < 0.001), particularly OSF stage 1 (p < 0.001) and stage 4 (p < 0.05). In OSCC, with and without OSF, the epithelial expression was more than connective tissue, but the difference was non-significant (p > 0.05). Overall, TGF-β1 immunoexpression was positive in OSF and OSCC with no significant difference (p > 0.05) and negative in NOM. In OSF, TGF-β1 increased from stage 1 to 3 but decreased in stage 4. OSF stage 3 had a significant increase compared to other OSF stages and OSCC with OSF (p < 0.05) (Figure 1).

TGF-β3 Expression in NOM, OSF (stage 1 to stage 4), and OSCC with and without OSF

In the epithelium, TGF-β3 expression was significantly low in OSF compared to OSCC without OSF (p < 0.001) and OSCC with OSF (p < 0.01). NOM and OSF had no significant difference. Stage 3 had a significant increase compared to stage 2 (p < 0.045) and stage 4 (p < 0.001), while stage 4 had a significant decrease compared to the other stages (p < 0.001). In connective tissue, TGF-β3 expression was significantly high in NOM and OSCC (with and without OSF) compared to OSF (p < 0.05). There was no significant difference between OSCC (with and without OSF) and NOM (p > 0.05) (Table 2).

**Table 2:** Comparison of TGF-β3 expression in epithelium and the connective tissue for NOM, OSF stage 1, OSF stage 2, OSF stage 3, OSF stage 4, and OSCC with and without OSF MW, Mann-Whitney U test; NOM, normal oral mucosa; K, Kruskal-Wallis test; OSCC, oral squamous cell carcinoma; OSF, oral submucous fibrosis; TGF-β3, transforming growth factor-beta 3 *Indicates statistical significance

Groups	Epithelium	Connective tissue	p-value
NOM	6.1 ± 1.07	6.55 ± 0.51	0.0932^MW^
	6 (2, 7)	7 (6, 7)	
OSF stage 1	6.05 ± 0.76	6.65 ± 0.59	<0.0094^MW^*
	6 (5, 7)	7 (5, 7)	
OSF stage 2	5.75 ± 0.91	5.85 ± 1.14	0.535^MW^
	6 (3, 7)	6 (2, 7)	
OSF stage 3	6.35 ± 0.81	6.5 ± 0.61	0.694^MW^
	7 (5, 7)	7 (5, 7)	
OSF stage 4	4.55 ± 1.57	5 ± 1.34	0.199^MW^
	5 (0, 7)	5 (0, 6)	
OSCC	6.5 ± 0.53	6.5 ± 0.71	0.863^MW^
	6.5 (6, 7)	7 (5, 7)	
OSCC + OSF	6.7 ± 0.48	6.5 ± 0.85	0.812^MW^
	7 (6, 7)	7 (5, 7)	
p-value	<0.001^K^*	<0.001^K^*	-

**Figure 2:**
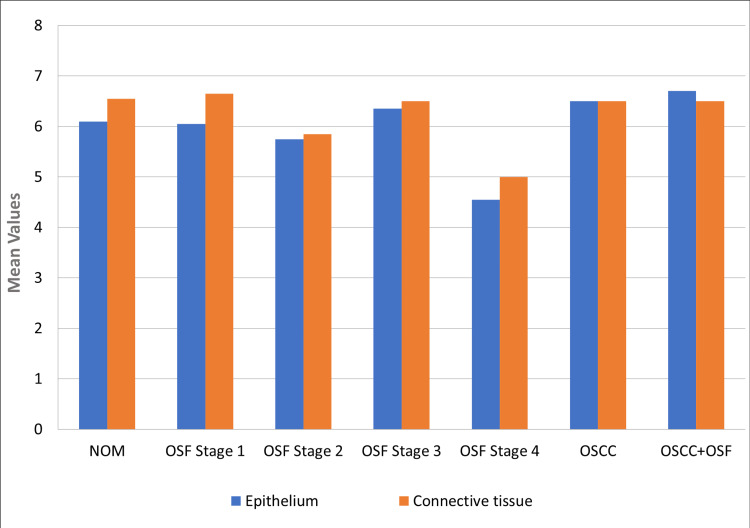
Mean quick score comparison of TGF-β3 expression in epithelium and the connective tissue for NOM, OSF stage 1, OSF stage 2, OSF stage 3, OSF stage 4, OSCC, and OSCC with OSF NOM, normal oral mucosa; OSCC, oral squamous cell carcinoma; OSF, oral submucous fibrosis; TGF-β3, transforming growth factor-beta 3

TGF-β3 expression was highest in OSF stage 1 and 3, followed by stage 2 and 4. A significant decrease was observed in OSF stage 2 and stage 4 compared to NOM, OSF stages 1-3, and OSCC with and without OSF (p < 0.05). Connective tissue expression was significantly higher than epithelium in all groups (p < 0.05), particularly in OSF stage 1 (p < 0.009). Overall, TGF-β3 expression was highest in OSCC, followed by NOM and OSF. There was a significant difference between OSF and OSCC with OSF (p < 0.05). TGF-β3 expression decreased significantly in OSF stage 4 compared to other stages and OSCC (p > 0.05) (Figure 2).

TGF-β1 versus TGF-β3 expression in NOM, OSF (stage 1 to stage 4), and OSCC with and without OSF

**Figure 3:**
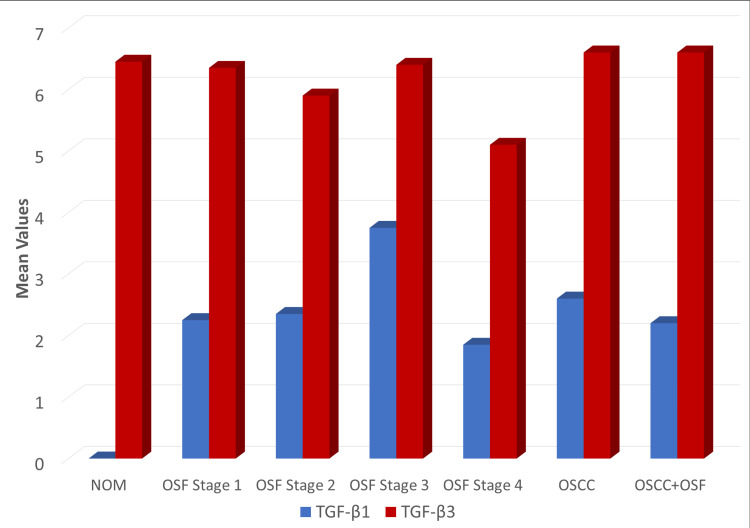
Mean quick score comparison of overall expression of TGF-β1 and TGF-β3 in NOM, OSF stage 1, OSF stage 2, OSF stage 3, OSF stage 4, OSCC, and OSCC with OSF NOM, normal oral mucosa; OSCC, oral squamous cell carcinoma; OSF, oral submucous fibrosis; TGF-β1, transforming growth factor-beta 1

TGF-β3 expression showed a highly significant increase in both epithelial and connective tissue compared to TGF-β1 in all groups (p < 0.001) (Table 3, Figure 3).

**Table 3:** Comparison of overall TGF-β1 and TGF-β3 expression of NOM, OSF stage 1, OSF stage 2, OSF stage 3, OSF stage 4, OSCC with and without OSF MW, Mann-Whitney U test; NOM, normal oral mucosa; K, Kruskal-Wallis test; OSCC, oral squamous cell carcinoma; OSF, oral submucous fibrosis; TGF-β1, transforming growth factor-beta 1; TGF-β3, transforming growth factor-beta 3 *Indicates statistical significance

Groups	TGF-β1	TGF-β3	p-value
NOM	0 ± 0	6.45 ± 0.51	<0.001^MW^*
	0 (0, 0)	6 (6, 7)	
OSF stage 1	2.25 ± 1.92	6.35 ± 0.67	<0.001^MW^*
	3 (0, 4)	6 (5, 7)	
OSF stage 2	2.35 ± 1.18	5.9 ± 0.85	<0.001^MW^*
	3 (0, 4)	6 (4, 7)	
OSF stage 3	3.75 ± 0.79	6.4 ± 0.68	<0.001^MW^*
	4 (2, 5)	6.5 (5, 7)	
OSF stage 4	1.85 ± 1.81	5.1 ± 0.72	<0.001^MW^*
	2 (0, 4)	5 (4, 6)	
OSCC	2.6 ± 2.07	6.6 ± 0.7	<0.001^MW^*
	3 (0, 6)	7 (5, 7)	
OSCC + OSF	2.2 ± 1.69	6.6 ± 0.7	<0.001^MW^*
	2.5 (0, 4)	7 (5, 7)	
p-value	<0.001^K^*	<0.001^K^*	-

## Discussion

In OSF, chronic sustained mechanical and chemical injury to the oral mucosa, mainly due to areca nut, triggers inflammation and tissue damage. According to researchers, OSF is an over-healing or a failed wound-healing process [2,10]. Tissue repair and fibrosis are closely linked, and TGF-β can aid in healing and contribute to fibrosis-associated disease [3,11,12].

TGF-β isoforms (TGF-β1, TGF-β2, and TGF-β3) share a high level of structural homology but exhibit differential spatial-temporal expression that results in biological outcomes that are quantitatively and qualitatively unique [3,6]. TGF-β1 promotes collagen production and fibrogenic response in multiple organs [3,11]. Anti-fibrotic activity of TGF-β3 has been studied in post‐myocardial infarction, human corneal fibroblasts, skin, lip, laryngeal mucosa [6,12,13]. Studies have shown that using TGF-β1 and TGF-β2 neutralizing antibodies and low concentrations of TGF-β3 within 48 hours of a wound injury can reduce fibrosis and scarring in rat cutaneous wounds. Increased TGF-β3 expression limits myofibroblast differentiation, which inhibits type I collagen synthesis and promotes degradation [6,12].

Extensive research has focused on the role of TGF- β1 in various organ fibrosis [3,5,11,12], but TGF- β3 has not yet received sufficient attention in the complex process of fibrosis. There are few studies on TGF- β1 [4,14-24] and only one on TGF-β3 in OSF [24]. Our study aimed to evaluate the possible role of TGF-β1 and TGF-β3 by IHC in normal healthy controls, OSF at different stages, and OSCC with and without OSF.

TGF-β1 in NOM, OSF, and OSCC

Our study demonstrated negative TGF-β1 immunoexpression in NOM samples, which aligns with few studies [14,15,22]. However, other studies have reported positive TGF-β1 expression in NOM [4,16,17,20,23,24].

**Figure 4:**
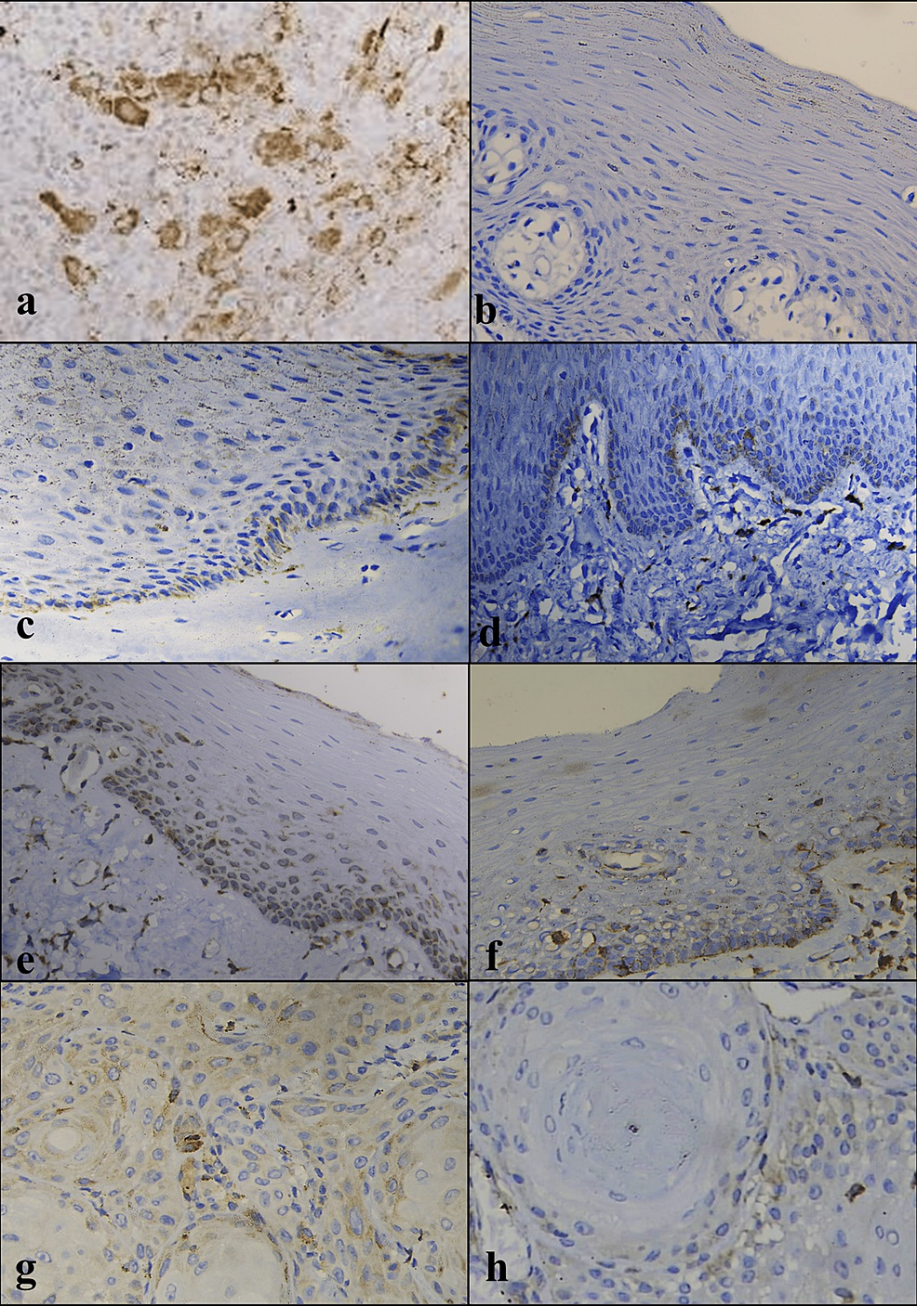
(a) Positive cytoplasmic expression of TGF-β1 in control – megakaryocytes, human spleen (objective 40X). (b) Negative expression of TGF-β1 in NOM (objective 40X). (c) Low positive expression of TGF-β1 in OSF stage 1 (objective 40X). (d) Low positive expression of TGF-β1 in OSF stage 2 (objective 40X). (e) Intermediate positive expression of TGF-β1 in OSF stage 3 (objective 40X). (f) Low positive expression of TGF-β1 in OSF stage 4 (objective 40X). (g) Intermediate positive expression of TGF-β1 in OSCC (objective 40X). (h) Low positive expression of TGF-β1 in OSCC+OSF (objective 40X). NOM, normal oral mucosa; OSCC, oral squamous cell carcinoma; OSF, oral submucous fibrosis; TGF-β1, transforming growth factor-beta 1

There was no significant difference in TGF-β1 immunoexpression among OSF and OSCC (with or without OSF); however, significant increase was noted in OSF stage 3 as compared to OSCC with OSF (Figure 4).

This finding indicates that OSF has the potential to become a malignant disorder and highlights the involvement of TGF-β1 in the malignant transformation of OSF. There was no significant difference between OSCC with OSF and without OSF, implying that both forms of OSCC have a common mechanism of carcinogenesis. Studies have shown higher TGF-β1 expression in OSCC [18,25,26], but no research has explored the association between OSF and OSCC. A study by Illeperuma et al. found no correlation between the expression of TGF-β1 and the degree of fibrosis or the grade of epithelial dysplasia in OSF [16].

Studies on OSF have reported that TGF-β1 positivity levels can range from 100% [4,16,20,23,24] to 60%-75% [14,19,21] with varying outcomes, including an increase in the early [19] or intermediate [20] or advanced OSF stage [16,23] or no discernible variations. One theory suggests that external stimuli (areca nut) and inflammation may contribute to higher TGF-β1 secretion in the early stages of OSF [19,21]. Our study found 75% (60/80 cases) TGF-β1 immunopositivity in OSF with a significant decrease from stage 3 to stage 4.

**Figure 5:**
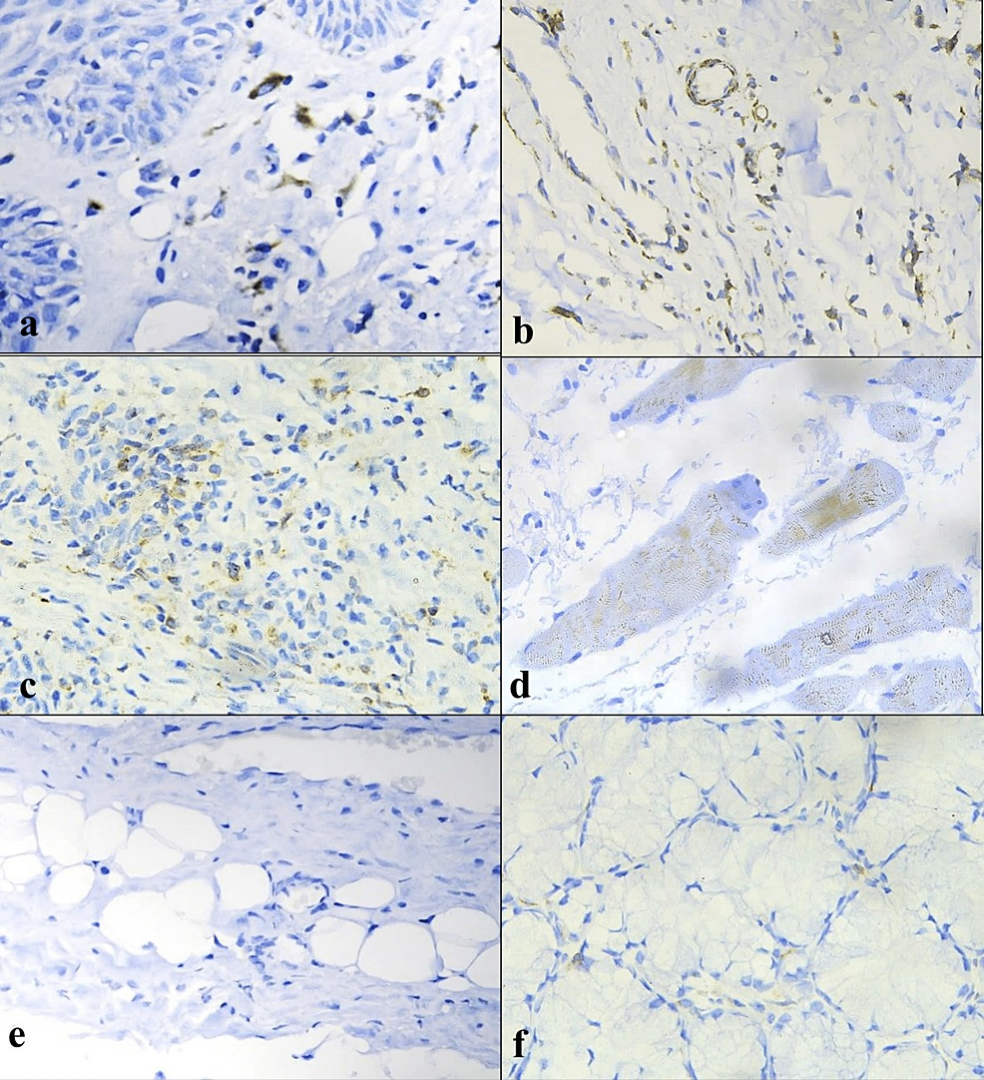
Positive TGF-β1 expression (objective 40X) in (a) fibroblasts, (b) blood vessels, (c) inflammatory cells, and (d) muscle. Negative TGF-β1 expression (objective 40X) in (e) adipocytes and (f) salivary gland. TGF-β1, transforming growth factor-beta 1

Our study found significantly higher TGF-β1 immunoexpression in OSCC (with and without OSF) compared to OSF in the epithelium. However, in the connective tissue, OSF had significantly higher levels than OSCC (with and without OSF), particularly in cases of OSCC with OSF. OSF stage 3 showed significantly high TGF-β1 expression. It is worth noting that while previous research has documented TGF-β1 expression in multiple layers in OSF [16,17,19,21,20], we only observed it in the basal layer followed by the spinous layer. Keratinocytes of OSF tissue produce and release TGF-β1, which may be an essential mediator in the pathogenesis of OSF [14,22]. TGF- β1 positivity was observed mainly in fibroblasts (62.5%) and inflammatory cells (28.3%), followed by muscle (9%) and endothelial cells (5%), while adipocytes and minor salivary gland tissue were negative (Figure 5).

Additionally, adipose tissue was absent in 33 (41.25%) of the 80 OSF samples (stage 1: 12/20; stage 2: 5/20; stage 3: 10/20; stage 4: 6/20), which could be due to increased TGF-β1 expression in OSF, resulting in lipodystrophy, as suggested by previous research [19].

TGF-β1 expression was also observed to be significantly higher in connective tissue than epithelium in OSF stages 1 and 4, indicating that TGF-β1 expression differs between the early and late phases of OSF. It also emphasizes TGF-β1’s critical role during fibrosis by inducing myofibroblastic transdifferentiation in mesenchymal cells. During the early stages, Pitiyage et al. found stronger TGF-β1 expression in OSF epithelium than in mesenchyme. They postulated that epithelial-produced active TGF-1 stimulates collagen synthesis in OSF fibroblasts via epithelial-mesenchymal transition [17].

TGF-β3 in NOM, OSF, and OSCC

**Figure 6:**
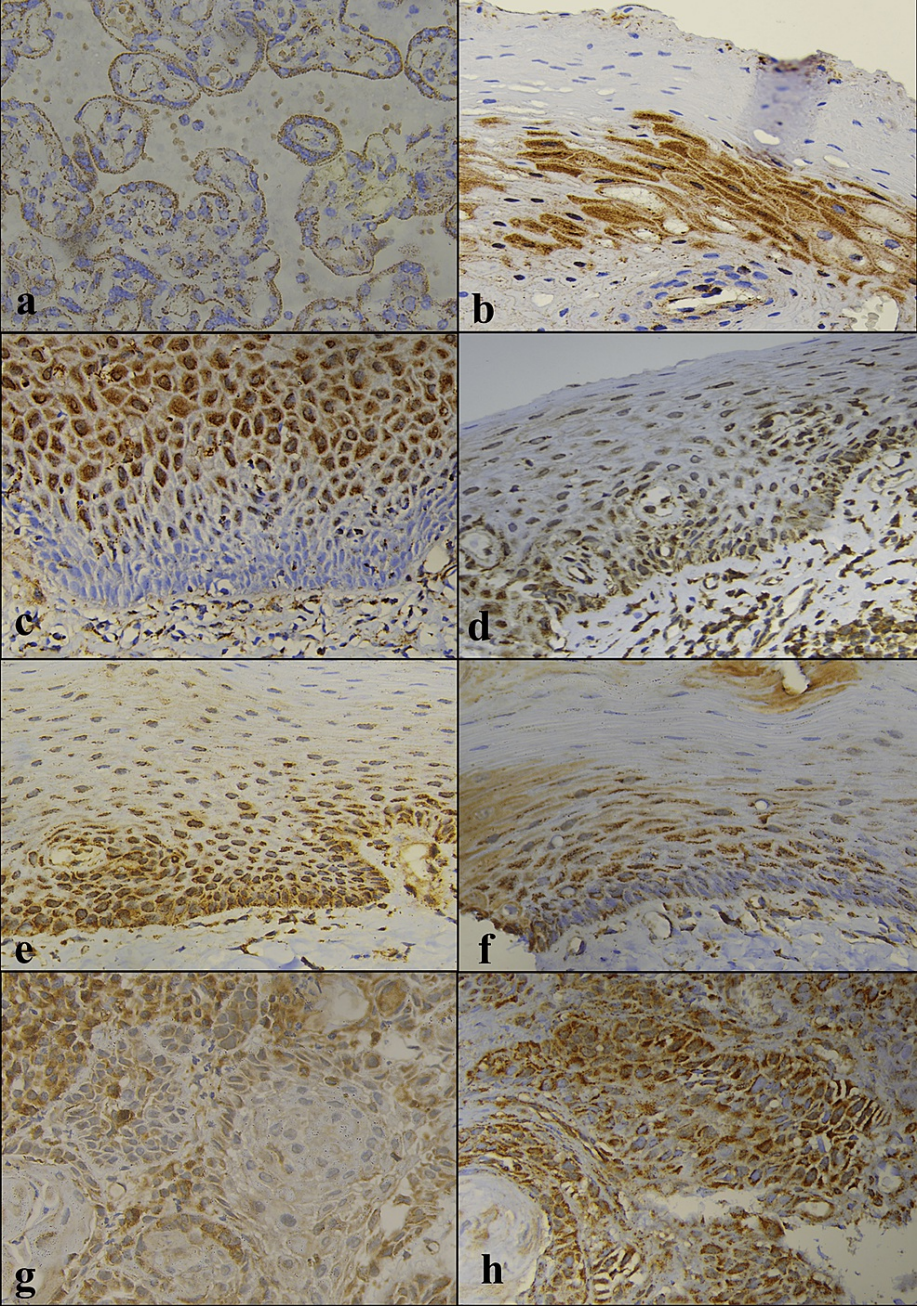
(a) Positive cytoplasmic expression of TGF-β3 in control - human placenta (objective 40X). (b) High positive TGF-β3 expression in NOM (objective 40X). (C) High positive expression of TGF-β3 in OSF stage 1 (objective 40X). (d) High positive TGF-β3 expression in OSF stage 2 (objective 40X). (e) High positive expression of TGF-β3 in OSF stage 3 (objective 40X). (f) Intermediate positive expression of TGF-β3 in OSF stage 4 (objective 40X). (g) High positive expression of TGF-β3 in OSCC (objective 40X). (h) High positive expression of TGF-β3 in OSCC+OSF (objective 40X). NOM, normal oral mucosa; OSCC, oral squamous cell carcinoma; OSF, oral submucous fibrosis; TGF-β3, transforming growth factor-beta 3

In the present study, TGF-β3 immunoexpression was highest in OSCC (with and without OSF) followed by NOM and OSF, with significant difference between OSF and OSCC (with and without OSF), more specifically OSCC with OSF (Figure 6).

There was no significant difference between NOM and OSF, as well as between NOM and OSCC (with and without OSF). In contrast to our findings, Rai et al. discovered significantly upregulated TGF-β3 expression in OSF compared to NOM using PCR, the only TGF-3 study on OSF [24]. Hussein demonstrated strong TGF-β3 positive expression in various stages of OSCC of the alveolar mucosa and tongue and concluded TGF-β3 as an effective diagnostic marker for identifying metastasis in OSCC [27]. We could not establish the clinical significance of our findings, as all patients with OSCC, regardless of whether they had OSF or not, were referred to a specialized center for further treatment.

Our findings indicate that changes in the dynamics of TGF-β3 expression in different stages of OSF contribute to the pathogenesis of OSF. TGF-β3 expression decreased from stage 1 to stage 2, increased in stage 3, and drastically decreased in stage 4. High TGF-β3 expression in stage 3 may suggest that the body's antifibrotic mechanism is activated, but it may be qualitatively and/or quantitatively insufficient to reduce fibrosis effectively. Significantly low TGF-β3 levels in stage 4 compared to NOM, OSF stages 1-3, and OSCC with and without OSF may demonstrate an essential role of TGF-β3 in preventing fibrosis during OSF progression.

**Figure 7:**
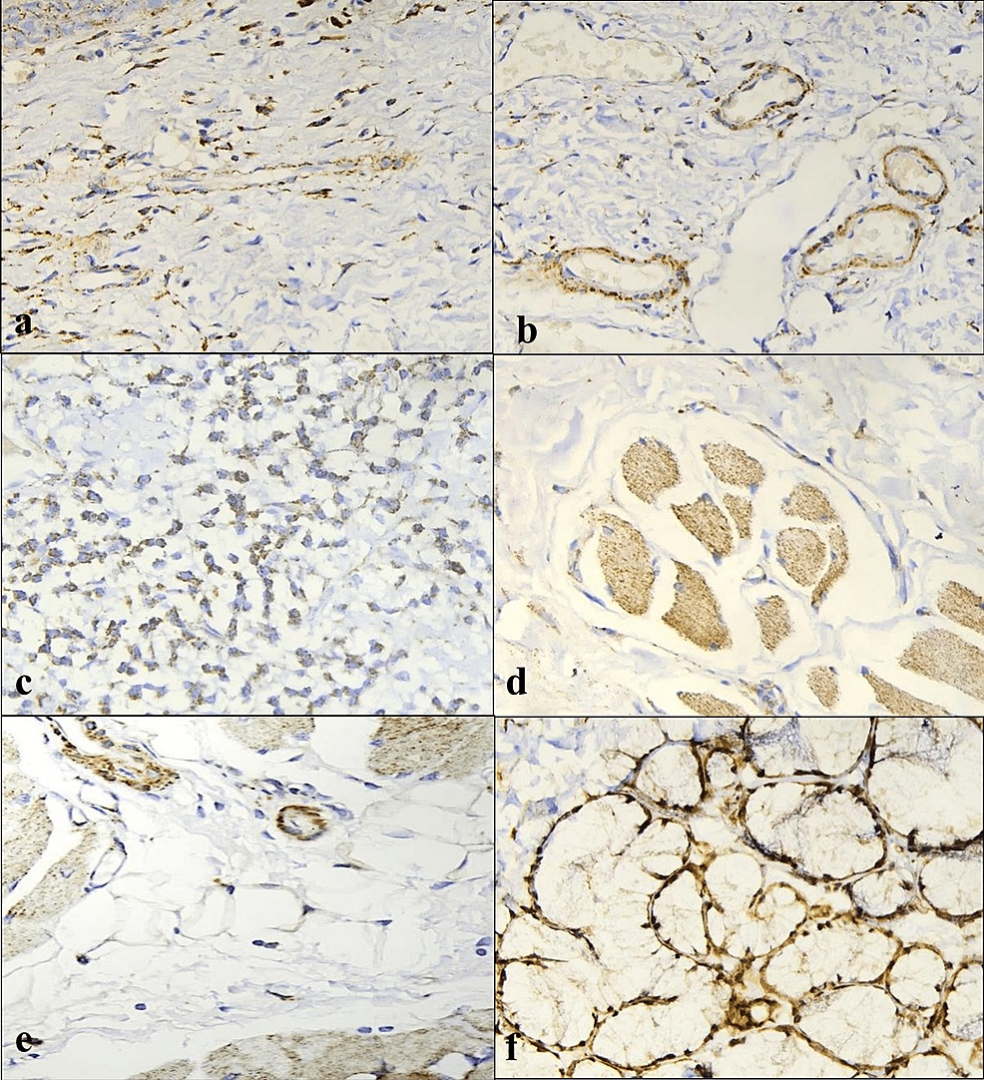
Positive TGF-β3 expression (objective 40X) in (a) fibroblasts, (b) blood vessels, (c) inflammatory cells, (d) muscle, (e) adipocytes, and (f) salivary gland. TGF-β3, transforming growth factor-beta 3

Further evaluation of TGF-β3 immunoexpression in the epithelium and connective tissue of all tissue samples revealed significantly low TGF-β3 expression in OSF than OSCC (with and without OSF) and non-significant difference among NOM and OSCC (with and without OSF). High TGF-β3 epithelial expression was seen primarily in the basal and spinous layer, followed by full-thickness involvement in a few cases. In OSF, high TGF-β3 expression was seen in fibroblasts (100%), inflammatory cells (100%), muscle (100%), endothelial cells (98.7%), followed by salivary gland tissue (60%), and adipocytes (19.1%) serving as potential sources of TGF-β3 in fibrosis (Figure 7).

Furthermore, we observed slightly high TGF-β3 expression in the connective tissue compared to the epithelium in NOM and OSF, and the difference was statistically significant in OSF stage 1. This demonstrates variation in TGF-β3 expression during the early stages of OSF in both epithelium and connective tissue.

Additionally, our results indicate that stage-specific expression of TGF-β1/TGF-β3 is distinct from overall OSF expression. It is vital to emphasize that OSF progresses from an inflammatory to an advanced fibrotic phase, which is irreversible and can result in malignancy. As a result, we recommend that anti-fibrotic therapy for OSF may be more effective if administered early in the disease to slow the fibrotic process.

TGF-β1 versus TGF-β3 in NOM, OSF, and OSCC

The expression of TGF-β3 was significantly upregulated in NOM, OSF, and OSCC (with and without OSF). This implies that while TGF-β3 levels were high, their levels may be insufficient to prevent or reduce fibrosis in OSF. Elevated TGF-β3 levels are more likely to be protective rather than causal in the development of OSF.

Gonçalves et al. reported similar findings in their study on TGF-β isoform expression in patients with oral leukoplakia, OSCC, and NOM. They observed high immunoexpression of TGF-β2 and β3, but not TGF-β1, in keratinocytes and stromal cells of OSCC and oral leukoplakia as compared to NOM [26]. Paterson et al. observed contradictory findings, revealing no significant differences in the staining intensity of TGF-β isoforms among NOM, primary squamous cell carcinomas, and metastatic lymph node tumor deposits [25].

A single study analyzed mRNA level of TGF-β1, TGF-β2, and TGF-β3 in NOM and OSF using polymerase chain reaction. They found a highly significant difference in the mRNA expression of TGF-β1 and TGF-β3 and a statistically significant difference of TGF-β2 between OSF samples and NOM. The TGF-β1 was the most expressed of the three TGF-β isoforms [24].

Role of TGF- β1 and TGF- β3 in OSF

Our study confirms previous research linking high levels of TGF-β1 to pathological tissue fibrosis, including OSF [3,4,11,12,14-24]. We found significantly elevated levels of TGF-β1 in OSF and absence in NOM, highlighting its pro-fibrotic effect in OSF. While insignificant, we observed slightly higher levels of TGF-β3 in NOM compared to OSF. Previous studies have shown TGF-β3 to be a potent inhibitor of fibrosis in various tissues, including the cornea, vocal fold mucosa, skin, and lungs, highlighting its protective role [5,6,13,12]. According to Xue et al., an increase in TGF-β3 concentration inhibits the proliferation and migration of human cardiac fibroblasts and reduces collagen synthesis in myocardial infarction [13]. We anticipate that a similar dynamic is at work in OSF, with higher levels of TGF- β3 attempting to alleviate fibrosis by counteracting increased collagen synthesis by TGF- β1.

As mentioned, TGF-β1 expression increased significantly from NOM to OSF, whereas TGF-β3 expression decreased from NOM to OSF. This indicates that despite having higher TGF-β3 levels than TGF-β1, the quantity or quality of TGF-β3 was lacking to prevent or reduce fibrosis in OSF.

Role of TGF-β1 and TGF-β3 in angiogenesis

TGF-β in endothelial cells can either promote blood vessel growth or hinder their maturation, depending on its expression levels and tissue context [11]. Our findings indicate that TGF-β1 is present in OSF tissue but absent in normal endothelial cells. This shows that TGF-β1 reduces angiogenesis in OSF by causing endothelial cell apoptosis, which can result in hypoxia in the surrounding tissue. Vascular endothelial growth factor increases as a compensatory response to maintain angiogenesis [28]. TGF-β1-induced endothelial-mesenchymal transition has been linked to various organ fibrosis [11]. We also observed the presence of TGF-β3 in both normal and OSF tissue. An in vivo study exposed chicken chorioallantoic membrane to recombinant TGF-β1, TGF-β2, and TGF-β3 and observed high angiogenic potency of TGF-β3, which can be beneficial in the treatment of ischemic and hypoxic wounds [29]. We believe that the coexistence of TGF-β3 in OSF helps maintain the vascular elements by counteracting the effects of TGF-β1-induced endothelial cell death.

Role of TGF-β1 and TGF-β3 in malignant transformation of OSF

TGF-β plays a pivotal role in tumor development and progression, but the contribution of its isoforms remains unclear. TGF-β1 plays a dual role: as a tumor suppressor in the early stages of cancer and as a tumor promoter in the later stages [3,6]. Many studies have shown high levels of TGF-β1 in cancer patients' plasma or tumor tissue [3,18,25-27]. Upregulated TGF-β1 expression is correlated with advanced-stage cancer and a reduced survival rate [30]. We found no significant difference in expression of TGF-β1 among OSF and OSCC with and without OSF samples, which may reaffirm OSF's potentially malignant status.

Currently, there is a dearth of functional data illustrating the causative role of TGF-β3 in tumourigenesis. Interestingly, studies have correlated upregulated TGF-β3 expression with protection against the onset of neoplasm and a better prognosis for this illness. According to one theory, the stroma of tumors behaves as "normal wound healing gone awry," activating the normal reparative process in the tumor's underlying tissue [6]. Our study shows that OSCC (with and without OSF), particularly OSCC with OSF, has a significantly higher level of TGF-β3 expression than OSF. Given that TGF-β3 plays a protective role in several cancers [6,25-27], as well as its significant role in scarless healing [5,9,11,13,29], it is reasonable to speculate that elevated levels of TGF-β3 expression in OSCC with OSF are a protective response to tumor "injury." OSCC occurring in the background of OSF is a clinicopathologically distinct entity with a better prognosis and oncological outcomes [31].

Research has shown that the development of fibrosis is vital for OSF's malignant transformation via TGF-β/SMAD (suppressor of mothers against decapentaplegic) canonical signaling and non-SMAD non-canonical signaling. Non-canonical pathways, including Rho GTPase (Rho), phosphoinositide-3-kinase (PI3K), mitogen-activated protein kinases (MAPK), and p53 are TGF-β-mediated pathways responsible for malignant transformation [3,5,6].

Our study has limitations such as errors in IHC processing, using archived tissue samples from different age groups, and a lack of a correlation between TGF-β1 and TGF-β3 expression and OSCC patient prognosis. It is recommended that future research employs advanced molecular techniques using genetically engineered mouse models and single-cell RNA sequencing to arrive at more conclusive results.

## Conclusions

Based on our research, we suggest that the molecular imbalance between TGF-β1 and TGF-β3 significantly affects the progression of OSF and its transformation into malignancy. We observed increased expression of TGF-β1 in OSF, which supports previous studies indicating its involvement in the pathogenesis of OSF. However, we also found higher levels of TGF-β3 in NOM, OSF, and OSCC compared to TGF-β1, which raises questions about its role in OSF. We speculate that the increased TGF-β3 levels in OSF could be a compensatory mechanism to counter elevated TGF-β1 and prevent or attenuate fibrosis in OSF patients. However, this increased TGF-β3 may be qualitatively or quantitatively insufficient to counter the fibrotic effect of TGF-β1 in OSF. Thus, we recommend focusing on isoform-specific investigations for molecularly targeted therapy instead of suppressing TGF-β indiscriminately to avoid adverse reactions and ensure effective treatment. Further research is needed to understand the role of TGF-β3 in OSF better.
